# Beta cell lipotoxicity in the development of type 2 diabetes: the need for species-specific understanding

**DOI:** 10.3389/fendo.2023.1275835

**Published:** 2023-12-08

**Authors:** Patricia Thomas, Meurig T. Gallagher, Gabriela Da Silva Xavier

**Affiliations:** ^1^ Centre for Systems Modelling and Quantitative Biomedicine, University of Birmingham, Birmingham, United Kingdom; ^2^ Institute for Metabolism and Systems Research, Birmingham Medical School, University of Birmingham, Birmingham, United Kingdom

**Keywords:** beta cells, lipotoxicity, type 2 diabetes, obesity, fatty acids, long-chain fatty acids

## Abstract

The propensity to develop type 2 diabetes (T2D) is known to have both environmental and hereditary components. In those with a genetic predisposition to T2D, it is widely believed that elevated concentrations of circulatory long-chain fatty acids (LC-FFA) significantly contribute towards the demise of insulin-producing pancreatic β-cells – the fundamental feature of the development of T2D. Over 25 years of research support that LC-FFA are deleterious to β-cells, through a process termed lipotoxicity. However, the work underpinning the theory of β-cell lipotoxicity is mostly based on rodent studies. Doubts have been raised as to whether lipotoxicity also occurs in humans. In this review, we examine the evidence, both *in vivo* and *in vitro*, for the pathogenic effects of LC-FFA on β-cell viability and function in humans, highlighting key species differences. In this way, we aim to uncover the role of lipotoxicity in the *human* pathogenesis of T2D and motivate the need for species-specific understanding.

## 1 Introduction

**Figure 1:**
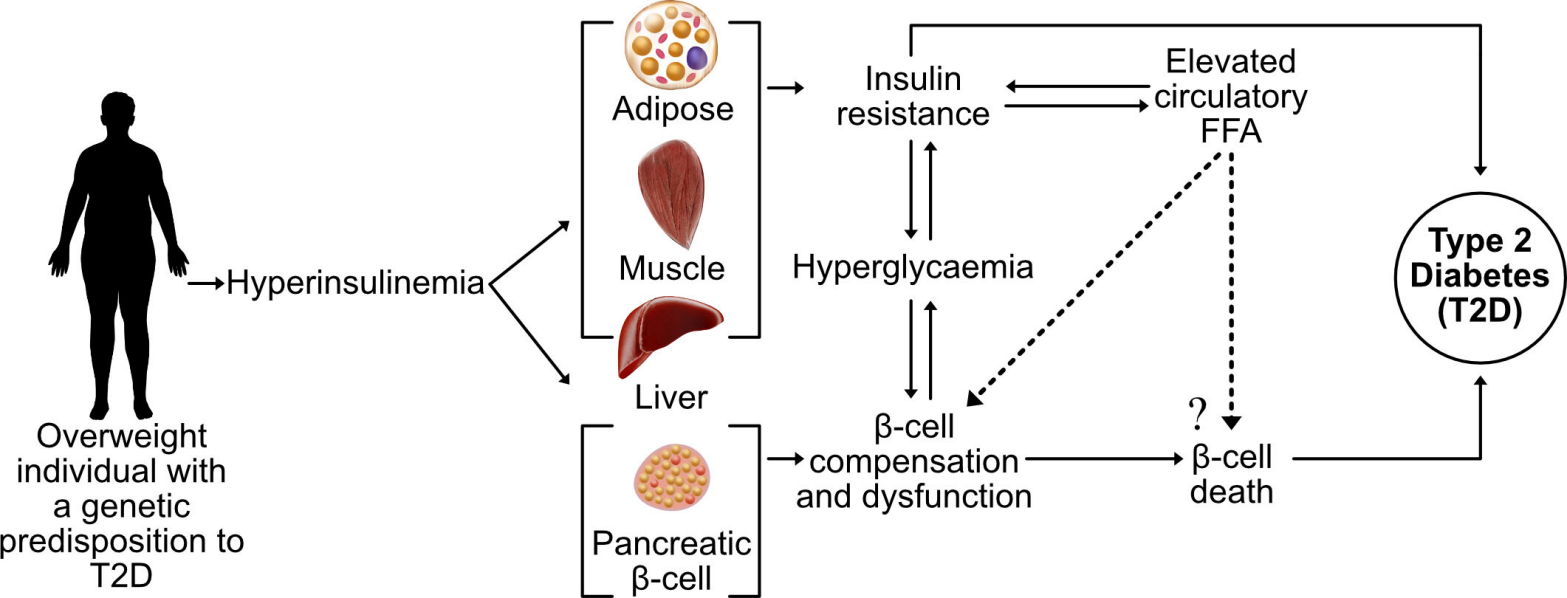
The current theory of β-cell lipotoxicity during the progression of T2D. A sedentary lifestyle coupled with a high-calorie diet leads to an accumulation of adipose tissue with hyperinsulinemia and elevated FFA, contributing towards insulin resistance of the peripheral tissue and hyperglycaemia. In genetically susceptible subjects, increased circulatory FFA concentrations are believed to contribute towards the death and dysfunction of insulin-producing pancreatic β-cells (lipotoxicity), leading to overt T2D.

Incidence rates of type 2 diabetes (T2D) have reached pandemic proportions, affecting more than 422 million individuals worldwide (1). The root cause of T2D is unknown, although it is well-established that obesity is the primary risk factor. The causal link between obesity and T2D remain unclear, but a feature of both conditions is an elevated blood concentration of long-chain free fatty acids (LC-FFA) (2). A decline in β-cell function and mass is the defining feature of T2D and it is widely believed that supraphysiological concentrations of circulatory LC-FFA are deleterious to β-cells through a process of lipotoxicity (**Figure 1**).

**Figure 2:**
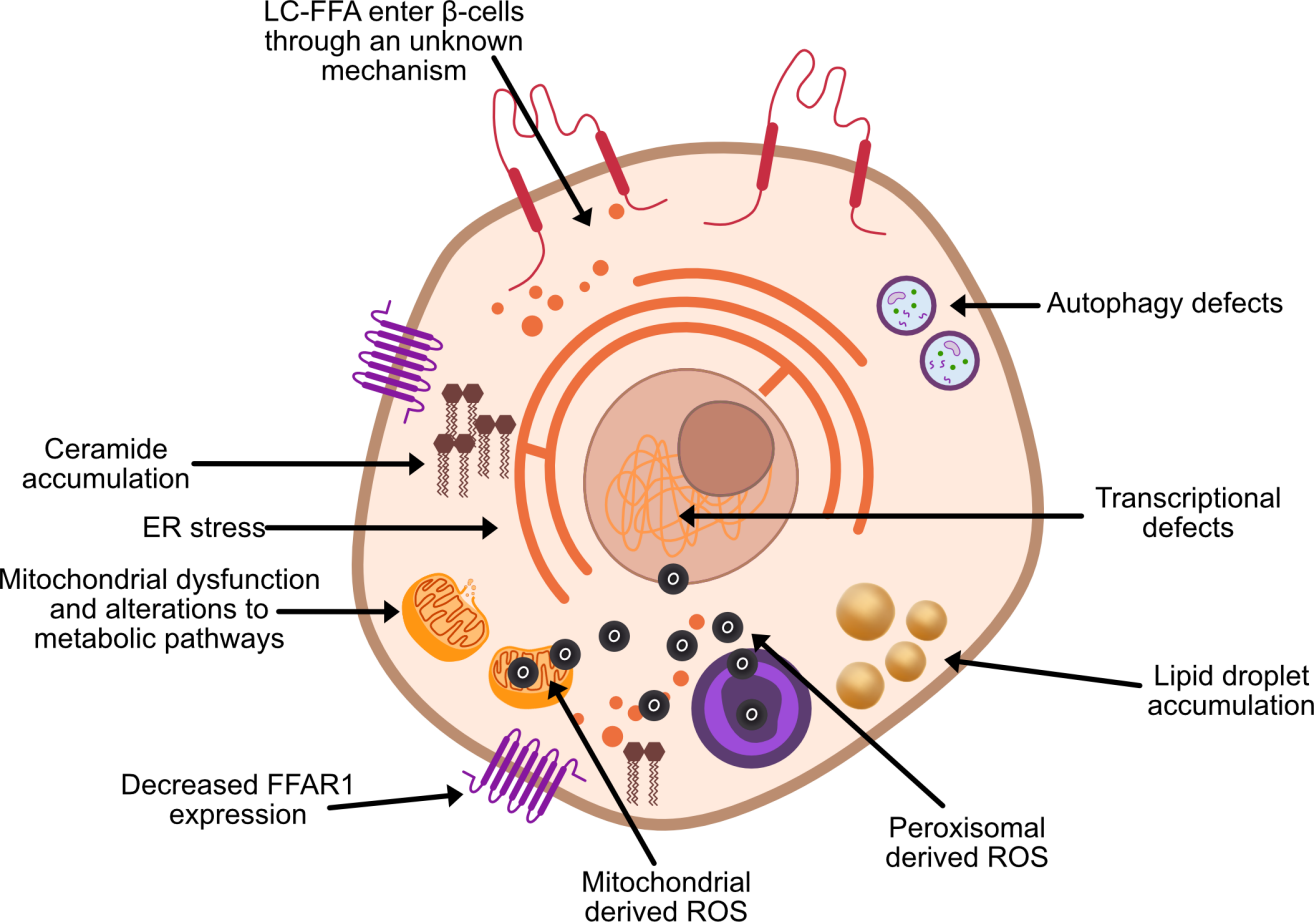
Proposed theories of β-cell lipotoxicity. ROS, reactive oxygen species; FFAR1, free fatty acid receptor 1.

Through their investigations into obese Zucker diabetic fatty rats, Unger et al. (3, 4) were among the first to suggest that chronically elevated circulatory FFA have a direct toxic effect on pancreatic β-cell function and viability. In subsequent years, considerable evidence has established long-chain saturated fatty acids (LC-SFA) species such as stearate and palmitate to be toxic to *in vitro* rodent β-cells upon chronic exposure [e.g (5)]. Extensive investigations have been undertaken to identify the mechanisms underpinning β-cell lipotoxicity (**Figure 2**), often with conflicting results.

Despite the popularity of lipotoxicity as a theory for the cause of β-cell dysfunction and death in T2D, the majority of literature that supports this concept is derived from rodent studies. Historically, difficulties isolating populations of β-cells from humans have slowed the study of human β-cell physiology (6). Rodent models have therefore played a key role in gaining a greater understanding of mechanisms underlying T2D, but there are significant differences between human to rodent β-cells. Such differences include the main glucose transporter (predominantly GLUT1 instead of GLUT2) (7), islet lipid handling (8, 9) and the sensitivity of human and rodent β-cells to the toxic effects of LC-SFA (10). It is not yet clear whether elevated circulatory LC-FFA concentrations occurring during the development of T2D are pathogenic *in vivo*. Delineating the effects of elevated circulatory FFA concentrations on β-cell function and viability remains a significant challenge, due to factors such as between-participant variability in plasma FFA levels/composition and β-cell secretory capacity through influences such as ethnicity, sex and genetic background.

There have been conflicting opinions regarding the theory of β-cell lipotoxicity, with strong voices both supporting (11) and opposing (12) it’s existence. The complexity of this topic, in part, lies in the amalgamation of data from both human and rodent models. As we show in this review, these two species have fundamentally differing responses to elevated levels of LC-FFA. This leads us to pose the question of how we develop species-specific understanding that clearly defines human pathology, enabling the targeted development of treatments in humans. In doing so, we review the evidence for a) the pathogenic effects of elevated LC-FFA on human β-cells *in vivo*, and b) human β-cell lipotoxicity *in vitro*. In this way, we aim to characterize what is currently known about T2D pathogenesis and highlight key areas where more data are needed to understand this disease.

## 2 Human β-cell lipotoxicity *in vivo*

Transportation of fatty acids throughout the body occurs via the blood, in either an esterified (predominantly as triglycerides) or non-esterified (as FFA) form, but it is the latter that are thought to be pathogenic in T2D (13, 14). Plasma FFA concentrations are tightly regulated between approximately 100-1000μM (15), but in samples from individuals with T2D, the FFA concentration has been shown to be 3-fold higher compared to age-matched controls (16). The source of surplus FFA in T2D is still unclear although it can in part be attributed to increasing *de novo* fatty acid synthesis, reduced FFA clearance, and enlarged adipocytes releasing more FFA [as reviewed by (17)].

*In vivo* studies assessing the relationship between FFA and human β-cell function report that high FFA concentrations are associated with a decline in insulin secretion (18–20), although this is not universally observed (21). It remains unclear whether this negative association is due to β-cell lipotoxicity or the adverse effects of elevated FFA concentrations on hepatic and peripheral tissue insulin sensitivity; with insulin resistance being a major feature of T2D. Measuring the deleterious impact of FFA on human β-cells *in vivo* remains challenging. In practice, we still do not know the precise concentration of FFA that β-cells are exposed to. Investigations that have sought to delineate the direct effect of excess FFA on human β-cells have largely been limited to exploring blood insulin levels only [e.g (20, 22)], which does not provide direct evidence for lipotoxicity *in vivo*. However, the majority of studies (8 for, 5 unclear, and 3 against; studies discussed below) identified in this review support that elevated plasma FFA are detrimental to human β-cells *in vivo*. This situation is mirrored in rodent studies (*in vivo*), where a controlled intravenous fat infusion causes a reduction in insulin secretion (23). Due to the complexity of the problem, a greater body of research is needed to gain a better understanding of *in vivo* human β-cell lipotoxicity. In what follows we aim to showcase and understand the progress made in this area so far.

### 2.1 The plasma free fatty acid profile and human β-cell lipotoxicity *in vivo*

Together with elevated concentrations of circulatory FFA, the plasma FFA profile has been shown to be associated with the development of T2D (24). In healthy lean individuals, the plasma FFA profile consists of fatty acids of more than 30 different species, with 78% of all FFA in circulation being comprised of palmitic (C16:0), stearic (C18:0) and oleic (C18:1) acid (25). As discussed by Sobczak et al. (26), those studies that have characterized the plasma FFA profile of individuals with T2D have found the data to be highly heterogeneous, although a common trend is an increase in the concentration of the LC-SFA palmitic and stearic acid (26).

In the EPIC-InterAct case-cohort study (27) elevated concentrations of the even-chained LC-SFA, palmitate (C16:0) and stearate (C18:0) were associated with an increased risk of developing T2D. Interestingly, elevated levels of the odd-chain LC-SFA, pentadecanoic (C15:0) and heptadecanoic acid (C17:0) were associated with a decreased risk (27). When testing the effects of pentadecanoic and heptadecanoic acid on the viability of the human β-cell line, EndoC-βH1, viability was maintained at high concentrations (500μM) following a 72h exposure period (10). However, viability was also maintained in EndoC-βH1 cells when exposed to palmitate and stearate, which are widely believed to be toxic to β-cell *in vitro* (10). In the RISC study cohort, raised levels of oleate correlated with enhanced β-cell function in non-diabetic individuals (22) indicating that oleate may not have the same deleterious effects as LC-SFA *in vivo*, supporting what has previously been shown *in vitro* (28). Although there is a clear relationship between T2D and obesity, many individuals with obesity do not develop T2D. Both conditions present with elevated concentrations of FFA in the blood, although individuals with prediabetes have been found to have a significantly greater concentration of plasma FFA compared to metabolically healthy obese subjects (29). Wrzosek et al. (2) found palmitic and stearic acid to be raised in both obesity and obesity-T2D, along with oleic and linoleic acid. However, the FFA profile was markedly different between the two groups (2) which could have pathogenic/diagnostic implications in T2D but requires further investigation. Not all studies support that the FFA profile is important. The PROMISE cohort found a predictor of lower β-cell function to be total FFA concentration, but not FFA profile (20).

Further investigations are required to understand the effect of changes in FFA composition on human β-cell function. To date, the data on changes to the blood FFA profile in T2D is heterogenous due in part, to a lack of appropriate matched controls (i.e. whether they were BMI matched), if the participants were fasted or not pre-blood collection, the ethnic group studied, and whether the results were reported as the percentage of total plasma FFA measured or absolute concentrations (26). In future studies, these factors need to be accounted for. Most investigations studying the effect of FFA on β-cells *in vitro* often only apply one or two LC-FFA at a time. Considering the broad array of FFA in circulation, and that different FFA species seemingly have different associations with T2D incident and effect on β-cell function, future *in vitro* studies should expose β-cells to a range of FFA to ensure physiological relevance.

### 2.2 Fat deposition and human β-cell lipotoxicity *in vivo*

The main storage site for FFA is in white adipose tissue (WAT) in the form of triglycerides (TAG). WAT is distributed subcutaneously (under the skin) or viscerally (around internal organs), and the site of WAT has distinct metabolic profiles (30). Ectopic fat is the storage of TAG in cells that are not adipose tissue, and which do not normally store large amounts of fat. Intrapancreatic lipid content has been shown to be inversely associated with insulin secretion and contributes towards β-cell dysfunction in the development of T2D (31, 32). Importantly, there are significant interindividual differences in the concentration of FFA that β-cells are exposed to, which result from differences in fat distribution due to ethnicity and sex (discussed in section 2.2.2 below).

#### 2.2.1 Intrapancreatic fat and human β-cell lipotoxicity

Magnetic resonance imaging (MRI) and spectroscopy (MRS) studies have revealed intrapancreatic fat content to be consistently raised in T2D subjects (31, 33–35). Although pancreatic fat is elevated in both obese non-diabetic and obese-T2D subjects, it is believed to be a feature independently related to T2D (34–36). Lu et al. (35) observed an association between pancreatic fat content and insulin secretion in male, but not female T2D subjects. In recent years, the work of Roy Taylor and colleagues has garnered significant attention for the proposal of “diabetes remission”.

Taylor et al. report a 14% decrease in body weight resulted in a significant fall in pancreatic fat in those individuals with T2D, but not in matched normal glucose tolerance individuals with comparable weight loss (34). Following a 15% weight loss over a 12-month period, a T2D cohort had a decrease in liver and pancreatic fat content coupled with the recovery of β-cell first-phase insulin response and glucose control (37). Whether the recovery of β-cell function was due to the decline in the liver or pancreatic fat however is unclear but the findings of this study (37) indicate that β-cells have the capacity to regain some function during the early stages of T2D progression. This is yet to be shown in longer-term studies and in a diverse ethnic cohort. Most studies that investigate the relationship between pancreatic fat content and T2D incident are cross-sectional, reporting only one point in time. There are few longitudinal studies investigating this question, only Yamazaki et al. (38) observed no independent association between pancreatic fat and T2D incident over a 5-year period (38).

Pancreatic fat may contribute towards the deterioration of human β-cell function in the early stages of T2D progression, although, there is limited evidence to support that it plays a role in the long-term development of T2D. To draw clearer conclusions, long-term studies are required on the recovery of β-cell function with sustained weight loss and loss of pancreatic fat content.

#### 2.2.2 The role of sex and ethnicity in understanding human β-cell lipotoxicity *in vivo*

**Figure 3:**
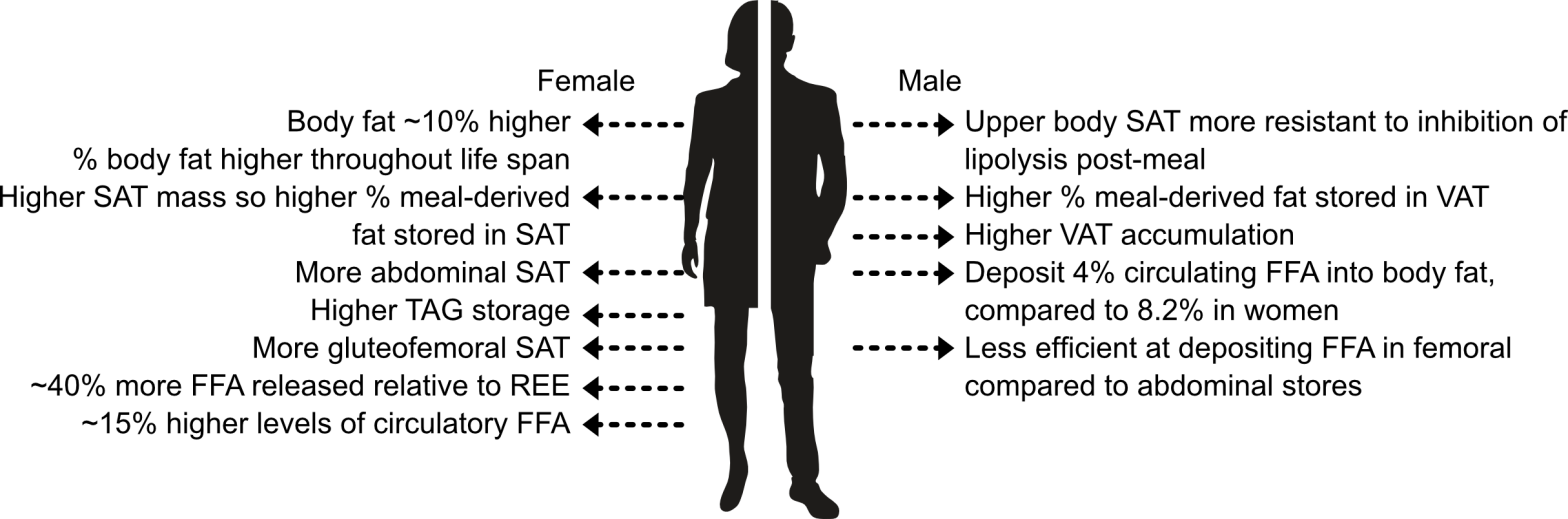
Key sex differences in fat storage. SAT, subcutaneous adipose tissue; REE, resting energy expenditure; TAG, triglycerides; VAT, visceral adipose tissue. Information extracted from (30).

In recent years, T2D has emerged to be more prevalent in men than women (39). As discussed by Kautzky-Willer et al. (40), psychosocial and biological factors can, in part, account for sex differences in T2D incidence and prevalence. A potential pathophysiological mechanism is differences in FFA metabolism and thus the concentration of FFA that the β-cells are exposed to. **Figure 3** shows key sex differences in fat distribution (for a more in-depth summary of these differences see (30).^1^.

Men and women store and use fat differently. Overall, women have a higher body fat percentage compared to men and store more fat in abdominal and gluteofemoral SAT (30), whereas men deposit more fat in VAT. It is believed that gluteofemoral SAT offers protection against T2D (41). Elevated levels of VAT are more harmful than SAT due to lipolysis releasing FFA which are transported through the portal vein directly to the liver contributing towards hepatic insulin resistance and steatosis (42, 43). Women tend to store FFA to a greater extent compared to men, as at rest and post-meal-consumption women are more likely to store FFA as TAG whereas men are more prone to oxidize the FFA [as discussed by (44)]. Insulin inhibits lipolysis; women have greater insulin sensitivity (45) causing differences in the rates of lipolysis between the sexes. Women have an approximately 15% higher concentration of circulatory FFA than men, and release approximately 40% more FFA relative to resting energy expenditure than men. This is thought to be due to women being more dependent on FFA oxidation during times of high energy requirements (e.g. exercise) (30). Subsequently, the β-cells of women may be exposed to higher concentrations of FFA although the concentration that β-cells are exposed to in either sex is currently still unknown.

There is also widespread acceptance that the prevalence and risk of developing T2D are higher amongst certain ethnic populations [as discussed by (46)]. Similarly to the disparities owing to sex, the reason for differences in T2D incidence rates amongst different ethnic groups is multifactorial, one of which could be differences in FFA metabolism between different ethnic populations (see **Supplementary Table 1**). As discussed by Goff (46), the genetic contribution of ethnic disparities in T2D incidence rate is unclear. However, differences in obesity and fat distribution across different ethnic groups are well-documented (47). For example, White European populations often develop T2D later in life and at a BMI of 30kg/m^2^, whereas South Asian populations develop T2D earlier and at a BMI of 22kg/m^2^ (48, 49). A reason for this may be due to the propensity of South Asian populations to store fat in abdominal VAT stores, less so than SAT, and have more ectopic liver fat compared to White Europeans causing greater metabolic complications (50). We have collated studies which have described fat distribution in high T2D-risk ethnic populations and investigated β-cell function (**Supplementary Table 1**).

In terms of β-cell lipotoxicity, it could be proposed that high-risk T2D populations may: 1) have higher circulatory concentrations of FFA and thus the β-cells are exposed to greater amounts of FFA; 2) have β-cells with greater sensitivity to lipotoxic insult. Goree et al. (51) found their female African American cohort to have lower basal fasting FFA compared to their European cohort. Ladwa et al. (52) found no association between intrapancreatic lipid content and insulin secretion in either a White European or Black African ancestry cohort. Collectively, this would indicate that high T2D risk ethnic groups are not exposed to more adverse concentrations of FFA compared to those at lower risk.

There are, however, disagreements in the literature. Szczepaniak et al. (32) found ethnic differences in both β-cell dysfunction and pancreatic fat. During a 20% intralipid infusion, Burns et al. (53) reported a comparable increase in fasting insulin and C-peptide concentrations in both Caucasian and African American adolescents. Conversely, Michaliszyn et al. (54) reported a decrease in β-cell function upon a 20% intralipid infusion in Caucasian and African American children which suggests that Caucasian youths may be more susceptible to lipotoxic insult. These results do not support the position that African American populations are more prone to developing T2D due to their β-cells being more sensitive to lipotoxicity.

In summary, these data imply that an alternative mechanism(s) to β-cell lipotoxicity may be contributing towards the higher risk of developing T2D in certain ethnic populations and between sexes. Differences in fat deposition between sexes and ethnic cohort groups seemingly contribute towards the risk of an individual developing T2D, with greater VAT and ectopic fat being adverse to metabolic health. Clearly the role of lipotoxicity between ethnic cohorts requires further investigation, as does determining the exact concentration of FFA that β-cells are exposed to *in vivo*. Importantly, when conducting ex vivo and *in vivo* studies using human islets, the sex and ethnicity of the participant/donor should be reported, and differences in FFA metabolism accounted for, as these are currently under controlled and will influence findings.

## 3 Human β-cell lipotoxicity *in vitro*

It is well documented that, upon acute exposure, LC-FFA can promote insulin release from human islets *in vitro* (55, 56), whilst chronic exposure impairs insulin secretion (54–56) and activates apoptosis (57–60). Conversely, human-derived EndoC-βH1 cells remain viable following chronic exposure to LC-FFA. This is potentially due to an elevated expression of the desaturase enzyme, stearoyl CoA desaturase (SCD) (61). LC-FFA may still induce dysfunction in this cell line; Jeffery et al. (62) found that when exposed to palmitate for 24h, EndoC-βH1 cells express somatostatin, a hormone that is selectively expressed by delta cells and not β-cells. This finding raises the question of whether high concentrations of LC-FFA may cause human β-cells to dedifferentiate, thus losing their identity and function – although the theory of β-cell dedifferentiation in T2D is currently a subject of research. Collectively these studies support that LC-FFA may cause human β-cell dysfunction *in vitro* and exploring the underpinning mechanism is the remaining subject of this review. In presenting this data, we have focused only on data taken from human islets or the human-derived EndoC-βH β-cell lines – with the latter being well characterized and insulin-producing (63). We omitted studies using 1.1B4 cells as they have been found to contain both rodent and human cells (64), and other human-derived β-cell lines (e.g., 1.4E7 or 1.1E7) due to their low insulin content (approx. 4ng/million cells) (65).

### 3.1 Fatty acid-induced changes to the transcriptome of human β-cells *in vitro*

Genome-wide association studies (GWAS) seek to find genetic variants that correlate with disease. GWAS has facilitated the identification of more than 128 common genetic risk variants for T2D (66) with palmitate modifying more than 11 GWAS candidate genes in human islets *in vitro* (57, 59, 67). RNA-sequencing analysis of human islets exposed to palmitate for 48h compared to non-exposed islets can promote more than 903 differentially expressed genes (DEG) (57, 59, 67) with the proportion of DEG increasing with prolonged exposure (67).

Functional analysis of DEG from human islets receiving an acute dose of palmitate (≤24h) show an enrichment of cellular pathways which may facilitate the augmentation of glucose stimulated insulin secretion (GSIS) (**Supplementary Table 2**); corresponding with a 2-fold increase in insulin secretion by islets treated for 24h with palmitate (67). Sargsyan et al. (67) propose that in the early stages of palmitate treatment, transcriptional changes give rise to both protective and deleterious cellular processes. With an acute treatment of palmitate protective cellular events outweigh deleterious processes, but the inverse occurs when palmitate exposure is prolonged (67).

With a chronic dose of palmitate (≥48h), DEG are associated with ER stress, inflammation, autophagy, protein degradation, metabolism and apoptotic pathways (57–60). Exposing islets to a chronic treatment of palmitate *in vitro* increases the expression of genes regulating LC-FFA metabolism (e.g. CPT-1 and ACSL1), but inhibits the expression of genes associated with the TCA cycle and electron transport chain (57). Thus, the loss of insulin secretion observed in islets treated with palmitate for prolonged periods (57, 68–70) may be due to impaired ATP synthesis. Cnop and colleagues found an inhibition of antiapoptotic genes (e.g. c-FLIP and ANXA4) and an increase in the mRNA for pro-apoptotic proteins, such as GRAMD4 which acts to inhibit antiapoptotic Bcl-2, and promote Bax translocation to the mitochondria in the initial stages of apoptosis (57). Following 48h palmitate treatment, there is also a downregulation of transcription factors (e.g. MAFA, MAFB, PDX-1 and NEUROD1) that regulate β-cell identity (57, 60). This suggests that LC-FFA may be a potential driver of β-cell dedifferentiation in T2D.

In summary, chronic exposure of high concentrations of LC-FFA induce widespread transcriptional changes which are deleterious to β-cell viability, identity, and function. However, the studies identified here (**Supplementary Table 2**) treat human islets with 0.5-1mM LC-FFA and only one FFA species. This raises the question of whether these conditions are physiologically relevant. LC-FFA studies regularly use a dosage of ≥0.5mM for lipotoxic conditions but it is still unclear what dose of LC-FFA are exposed to *in vivo*. Further, β-cells are exposed to varying LC-FFA in the blood. Future studies should therefore assess changes to the transcriptome following the application of a LC-FFA mix.

### 3.2 The role of lipid droplets in human β-cell lipotoxicity

In recent years, lipid droplets (LD) have emerged as a dynamic organelle that play a critical role in cellular lipid metabolism. LDs are composed of a neutral lipid core surrounded by a phospholipid monolayer. Lipids are released from the LDs for signaling, phospholipid synthesis, fuel and can even act to sequester harmful lipid intermediates. However, it remains unclear whether LDs are negatively or positively associated with the demise of human β-cells in the development of T2D.

Unlike in mouse β-cells, LDs and their associated proteins are enriched in human β-cells exposed to an exogenous source of LC-FFA (71, 72). In the regulation and turnover of LDs in human β-cells, adipose triglyceride lipase (ATGL) has been found to be a key lipase for LD mobilization, with its silencing increasing the number and size of LDs (73). Thomas et al. (9) showed that contrary to rodent β-cells, LC-SFA are trafficked into LDs in human EndoC-βH1 β-cells which may offer an explanation for why, unlike rodent β-cells, human β-cells are resistant to the toxic effects of LC-SFA. Further, Tong et al. (74) found that LDs preserve fatty acid homeostasis, thereby proving essential for human β-cell activity. Collectively, this would suggest that LDs may be a previously unrecognized organelle that protects human β-cells from the lipotoxic effects of LC-FFA. However, Tong et al. (8) observed an accumulation of LDs in β-cells from donors with T2D whereas visibly fewer LDs were present in healthy donors and that LD number increases with age. Whether LDs may be a protective or deleterious organelle during the development of T2D requires further investigation. Crucially, β-cell LDs are poorly characterized and elucidating their role in β-cell dysfunction and viability should be the subject of future work.

### 3.3 Fatty acid-induced endoplasmic reticulum stress in human β-cells

**Figure 4:**
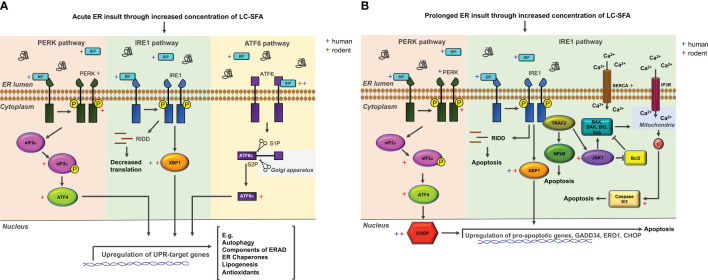
The ER stress network and members activated in rodent and human β-cells exposed to LC-FFA. **(A)** Within the ER lumen, misfolded and unfolded proteins accumulate, sequestering and binding to BIP, which triggers the activation of PERK, IRE1, and ATF6. ER stress sensors are then activated, initiating the UPR pathway which, downstream results in the upregulation of genes that alleviate ER stress. **(B)** With prolonged ER stress, ER stress sensor activation initiates predominantly the PERK- and IRE1-dependent ER stress pathways to induce an apoptotic response. Activating transcription factor 4 (ATF4); Ccl-2 homologous antagonist/killer (BAK); Bcl-2-associated X protein (BAX); BH3 interacting-domain death agonist (BID); Immunoglobulin heavy-chain binding protein (BIP); Cytochrome c (C); CCAAT-enhancer-binding protein homologous protein (CHOP); eukaryotic translation initiation factor 2α (eIF2α); inositol 1,4,5-triphosphate (IP3R); inositol requiring ER-to-nucleus signal kinase 1 (IRE1); c-Jun N-terminal kinase (JNK1); Nuclear factor ĸB (NFĸB); PKR-like ER kinase (PERK); IRE1 dependent decay (RIDD); SH_3_ homology-associated BTK binding protein (Sab); sarco/endoplasmic reticulum Ca^2+^-ATPase (SERCA); site-1 protease (S1P); TNF receptor-associated factor 2 (TRAF2); X-box binding 1 (XBP1). Information of potential mechanisms extracted with reference to (76–79).

For pancreatic β-cells, the endoplasmic reticulum (ER) is an essential organelle. More than 50% of protein production is insulin and the need for β-cells to raise by several folds their insulin biosynthesis in response to rising glucose concentrations poses a major challenge for the ER (75). Disruption to the homeostasis of the ER by pharmacological and physiological stressors can trigger ER stress. This process causes unfolded or misfolded proteins to gather at the ER, activating stress sensors that induce the unfolded protein response (UPR). The UPR is mostly initiated for the restoration of ER function, although it can perform the role of a binary switch between cell death and survival. Acute ER stress (**Figure 4A**) activates a regulated UPR, promoting cell survival, whereas prolonged ER stress (**Figure 4B**) causes UPR hyperactivation leading to apoptosis (80).

Immunostaining for ER stress markers provides evidence that this network is active in ex vivo human islets of T2D donors (81). While there is a wealth of evidence linking lipotoxicity to ER stress in rodent β-cells [e.g. (27, 82–84)], fewer studies support FFA-induced ER stress in human β-cells (28, 85). In rodent β-cells, palmitate can activate the expression of 9 ER stress markers within the PERK, IRE1 and ATF6 pathways, impair ER Ca^2+^ stores and trigger ER-stress-induced apoptosis (**Figure 4A, B**). Those studies that have investigated LC-SFA induced ER stress in human islets have found palmitate to activate 4 ER stress markers across the 3 arms of the chronic ER stress pathway (**Figure 4A, B**). Exposing human islets to 500μM palmitate for 24h significantly increased the expression of BIP, CHOP, and PERK compared to control-treated islets, and caused ultrastructural changes to the ER, increasing volume density (85). Exposing palmitate to human islets for 48h also increased the expression of ATF3, CHOP, XBP1s and BIP (28). Similar to findings in rodent models, LC-MUFA oleate did not induce ER stress signaling in human islets (28). It is believed that under certain conditions, lipotoxicity can be exacerbated by elevated glucose concentrations (glucolipotoxicity). The ER stress markers ATF3 and CHOP were upregulated in human islets in a glucose-independent manner and glucose failed to elicit a response independently (28).

In β-cells derived from human embryonic stem cells (SC-β-cells), palmitate treatment increased the expression of ER-stress markers IRE1α, XBP1 and sXBP1 (86). Strikingly, ZnT8 loss of function attenuated palmitate-induced ER stress in SC-β-cells via modulation of zinc levels (86). Conversely, palmitate alone and palmitate in combination with high concentrations of glucose did not induce ER-stress in EndoC-βH1 cells (9, 63). This may be a feature of the EndoC-βH1 cell line. Oleson et al. (87) failed to elicit ER-stress when treating EndoC-βH1 cells with the Ca^2+^ ATPase (SERCA) inhibitor, thapsigargin. This was attributed to the basal expression of heat shock protein 70 (HSP70) protecting EndoC-βH1 cells against ER stress activators (87). Other labs have however reported thapsigargin to induce ER stress in EndoC-βH1 cells, with Cunha et al. reporting thapsigargin to trigger EndoC-βH1 cell death (9, 87, 88). To summarize, there is evidence to support LC-FFA induced ER stress in human β-cells although this pathway has mainly been characterized in rodents. Future studies should characterize the ER stress pathway in human β-cells upon lipotoxic insult.

### 3.4 Fatty acid-induced impaired autophagy in human β-cells

Macroautophagy (autophagy hereafter) is the main intracellular degradation pathway. Autophagosomes sequester cytoplasmic material (e.g., lipids) prior to fusing with lysosomes where their content is degraded. The degraded material is then released back into the cell, thereby providing new building blocks or a source of energy. Autophagy contributes towards maintaining cellular homeostasis but under stress conditions, it can mediate cell death or survival. Emerging evidence suggests that autophagy is impaired in β-cells of T2D donors (87). Rodent β-cells incubated with LC-SFA show alterations to autophagy (89–91). However, in the rodent data, a consensus is lacking on whether β-cell autophagy is protective or deleterious upon lipotoxic insult, and if LC-SFA increases or impairs autophagic flux.

Autophagy can be stimulated in human islets through LC-FFA exposure (91, 92). Although, few studies have explored whether autophagy contributes towards human β-cell lipotoxicity. Despite this paucity of investigations, overloaded autophagosomes coupled with a reduction in the expression of the lysosomal proteases cathepsin B and D, and lysosome-associated membrane protein 2 (LAMP2), have been reported in β-cells from subjects with T2D (93). Cathepsin B and D and LAMP2 are involved with lysosomal fusion and protein degradation, respectively. Exposing human islets for 24h to 1mM LC-FFA (oleate/palmitate 2:1) triggers β-cell death, vacuole accumulation and a decrease in LAMP2 expression (93). This implies that in T2D, there may be a reduction in β-cell autophagic flux and if lipotoxicity is the cause, potentially through an obstruction in lysosomal fusion. There is also evidence to support that autophagy plays a protective role against ER stress in human β-cells (85). Under conditions of palmitate-induced ER stress, rapamycin, a known inducer of autophagy, prevents the expression of ER stress markers and apoptosis in human islets (85). The autophagy inhibitor, 3-MA, also enhances palmitate-induced human β-cell apoptosis (85). Collectively, these preliminary studies support the contribution of autophagy to human β-cell lipotoxicity; however it remains to be determined whether autophagy is deleterious or protective and this should be the subject of future studies.

### 3.5 Fatty acid-induced mitochondrial dysregulation in human β-cells

It is likely that mitochondrial aberrations contribute towards β-cell lipotoxicity, as this organelle plays a prominent role in both insulin secretion and apoptotic pathways. Mitochondria are complex organelles that constantly undergo a process of fission and fusion to form intracellular networks for the distribution of metabolites, proteins and lipids to facilitate metabolic efficiency.

Perturbations to mitochondrial morphology and dynamics can have profound effects on insulin secretion and can instigate apoptosis in rodent β-cells (94, 95); a process that can be stimulated through LC-SFA exposure (95). Located in the inner mitochondrial membrane is the non-specific, Ca^2+^-dependent, mitochondrial permeability transition (MPT) pore. It is widely understood that palmitate promotes the opening of the MPT, causing mitochondrial swelling and protein release which can lead to apoptosis (96). Also located at the inner mitochondrial membrane is the apoptogenic factor, cytochrome c, anchored by the phospholipid, cardiolipin. Cardiolipin remodeling through the incorporation of saturated fatty acid species can stimulate the dissociation of cytochrome c, triggering the apoptotic pathway (97). However, LC-FFA-induced mitochondrial remodeling, swelling and disruption to networks has, to the best of our knowledge, been shown almost exclusively in rodent β-cells.

In β-cells from T2D donors, there is a similar number of mitochondria relative to control subjects, however, the mitochondrial volume density is significantly higher with an increased protein expression of UCP-2, complex I and V of the electron transport chain (85, 98). Islets from individuals with T2D have an increased mitochondrial density with lower cytoplasmic ATP levels, a lower ATP/ADP ratio and an impaired yperpolarization of the mitochondrial membrane which impacts the insulin secretory response to glucose (98). Collectively, these observations support that mitochondrial dysfunction (particularly the MPT theory) is present in human β-cells although further investigations are required to establish if lipotoxicity is the cause.

### 3.6 Fatty-acid induced oxidative stress in human β-cells

An excessive accumulation of reactive oxygen species (ROS) coupled with an insufficient antioxidant response can result in oxidative stress. Pancreatic β-cells lack a comprehensive antioxidant system and thus are susceptible to oxidative stress, potentially due to ROS acting as a metabolic signaling molecule for GSIS in this cell type (99). However, a sustained level of ROS can cause lipid peroxidation, DNA damage, and the oxidation of proteins, which can cause β-cell death. The principal ROS identified in β-cells includes hydrogen peroxide, superoxide and hydroxyl radicals, which are mostly derived from the oxidation of LC-FFA (100). Peroxisomes produce ROS as a bi-product of FFA β-oxidation and mitochondrial FFA oxidation produces ROS through complexes I and III of the electron transport chain (101). Thus, the theory of β-cell lipotoxicity involves enhanced ROS formation by peroxisomal and mitochondrial FFA oxidation, coupled with a poor antioxidant system thereby contributing towards β-cell death.

In rodent β-cells, topics of debate include whether mitochondrial or peroxisomal-derived ROS has the greater contribution towards β-cell lipotoxicity (101). Although it has previously been shown that mitochondrial-derived ROS can cause rodent β-cell death (through mitochondrial DNA damage) (102), uncoupling protein-2 (UCP-2) may act as a protective mechanism. UCP-2 acts to uncouple the electron transport chain when there is a surplus of LC-FFA, thereby decreasing ATP production via the lowering of the mitochondrial membrane potential. The activation of UCP-2 is believed to stop ROS production and enable the export of harmful peroxides from the mitochondrion (103). However, the UCP-2 theory is debated (104) and a reduction in ATP production due to LC-FFA-induced UCP2 activity may still have a negative impact on insulin secretion.

Oxidative stress markers (such as 8-OH-deoxyguanine) have been observed in pancreatic biopsies from individuals with T2D (105, 106). In islets of T2D subjects there is also an increased expression of UCP-2 at the protein level, and higher levels of nitrotyrosine compared to non-diabetic controls (98, 107). Human islets exposed to LC-FFA for a 24h period have an accumulation of the nitrogen-free radical biomarker, nitrotyrosine, which was reduced with the application of the antioxidant, IAC (107). Oxidative stress markers are coupled with a change in the gene expression of enzymes involved in ROS scavenging, including a reduction in SOD1 and an increase of HO-1, glutathione peroxidase and catalase (107, 108). Collectively, this supports that LC-FFA have the ability to alter the antioxidant system of human β-cells but more research is needed to clarify this effect and should be the topic of future work.

## 4 Conclusion

Due to the complexity of the problem, and the lack of conclusive evidence, it is unclear whether β-cell lipotoxicity occurs in human pathology *in vivo*. As we have shown in this review, this is complicated by the lack of consensus regarding how changes in blood FFA composition and concentration are reported, and which key factors were accounted for when creating matched controls. While science should not aim to be too prescriptive, it may be that the development of consensus guidance, akin to a ‘core outcome set’ often seen in clinical trials, could be beneficial. Such a document would aid in the standardization of reporting of data for determining the effects of FFA on β-cell function *in vivo*, thereby allowing for greater insight when aggregating results.

It is clear from our review of the evidence that further *in vivo* studies are required to establish the role that LC-FFA play in the demise of β-cells during the development of human T2D. However, there is a wealth of evidence to support that chronic exposure of LC-FFA to human β-cells *in vitro* is deleterious (see **Table 1**). It is pivotal, however, that the exact concentration of LC-FFA that the β-cells are exposed to is determined as without this knowledge it cannot be inferred whether lipotoxicity is relevant to the human condition. Similarly, as LC-FFA can exert different physiological effects on human β-cells future studies should use a range of LC-FFA species within their investigations.

**Table 1:** Key differences in β-cell lipotoxicity between humans and rodents.

Theory of β-cell lipotoxicity	Human (β-cells)	Rodent (β-cells)
Lipid homeostasis	LC-SFA trafficked to lipid droplets (8, 9, 73).	LC-SFA not trafficked to lipid droplets (9).
ER stress	LC-SFA activate the expression of 4 ER stress markers (27, 84).	LC-SFA activate the expression of 9 ER stress markers (26, 81–83).
Impaired autophagy	Markers of impaired autophagy in pancreatic sections from T2D donors (85, 91).	Rodent β-cells treated with LC-SFA have alterations in autophagy (87–89) but unclear whether LC-SFA increases or impairs autophagic flux.
	Human islets treated with LC-SFA have overloaded autophagosomes with a potential reduction in lysosomal fusion (91).	
Mitochondrial dysregulation	In β-cells from individuals with T2D: Increased mitochondrial density (96). Increased UCP-2 expression (96). Impaired hyperpolarisation of the mitochondrial membrane (96). Uncertain whether lipotoxicity is the cause.	LC-SFA induce mitochondrial permeability transition pore activation leading to rodent β-cell apoptosis (94).
		LC-SFA induce cardiolipin remodelling leading to rodent β-cell apoptosis (95).
Oxidative stress	Markers of oxidative damage found in pancreatic biopsies from T2D individuals (103, 104).	Unclear whether ROS (produced as a bi-product of LC-SFA breakdown) from the mitochondria or peroxisomes play a greater role in rodent β-cell lipotoxicity (99).
	Treating β-cells with LC-SFA causes nitrogen free radicals to accumulate and a change in the expression of ROS scavenger proteins (105, 106).	UCP-2 may act as a protective mechanism against mitochondrial derived ROS (101).
Inflammation	Islets from individuals with T2D display pro-inflammatory mediators (79, 109).	LC-FFA induces pro-inflammatory factors in rodent β-cells (109).
	LC-SFA increase the expression of pro-inflammatory markers in non-diabetic β-cells (79, 109).	LC-SFA activates the STING-IRF3 (stimulator of interferon gene stimulator and interferon regulatory factor 3) signalling pathway which initiates inflammatory and apoptotic pathways in rodent β-cells (110, 111).
	Proinflammatory cytokines can trigger human β-cell death (10).	

## Author contributions

PT: Conceptualization, Funding acquisition, Writing – original draft. MG: Writing – review & editing. GS: Writing – review & editing.

## Footnotes

1 Here we use (wo)men to mean natal (wo)men (i.e. those assigned (fe)male at birth).
