# Supplementary material for: Beta cell lipotoxicity in the development of type 2 diabetes: the need for species-specific understanding
Source: Front Endocrinol (Lausanne). 2023 Dec 8;14:1275835. doi: 10.3389/fendo.2023.1275835 (PMC10739424; doi:10.3389/fendo.2023.1275835)
Supplement: Supplementary file 1 [file Table_1.docx]

| **Supplementary Table 1. Summary of studies into fat metabolism and beta cell function in different ethnic cohorts** | | | | |
| --- | --- | --- | --- | --- |
| Ref. | Sex and age | Ethnicity* | Metabolic status | Main Findings |
| Ladwa et al. (2021) (1) | Male (n=46)  18-49yrs old | Black African ancestry (n=23),  White European ancestry (n=23) | ND | Intrapancreatic lipid was not a predictor of insulin secretion in either ethnic group.  Postprandial insulin secretion was higher in the white European cohort compared to the black African ancestry cohort. |
| Trico et al. (2019) (2) | Males (n=549), Females (n=685)  35-53yrs old | European | ND | Circulating palmitoleate correlates with β-cell function |
| Michaliszyn et al. (2013) (3) | Males (n=11), Females (n=14) 9-10yrs old | African American (n=12), Caucasian (n=13) | Family hx of T2D (n=13)  No family hx of T2D (n=10) | β-cell function relative to insulin sensitivity decreased with a 20% intralipid infusion in both the African American and Caucasian cohort, with a significantly greater decrease in the Caucasian group. |
| Tillin et al. (2013) (4) | Male (n=1755) 43-59yrs old | European (n=1052), Indian Asian (n=703) | ND (n=1351), T2D (n=404) | Indian Asian cohort were 3x more likely to develop T2D compared to European cohort.  Indian Asian cohort with T2D have a lower BMI but higher central adiposity compared to European cohort.  Indian Asian cohort had higher fasting insulin and higher β-cell function compared to European cohort in both ND and T2D groups. |
| Anand et al. (2011) (5) | Male (n=53), Female (n=55) 31-39yrs old | South Asian (n=56), European (n=52) | ND | South Asian cohort had greater body fat mass compared to White Caucasian cohort.  South Asian cohort had less abdominal SAT, more VAT and ectopic liver fat compared to White Caucasian cohort.  South Asian cohort had higher fasting insulin compared to White Caucasian cohort (attenuated when adjust for fat distribution) |
| Lê et al. (2011) (6) | Males (n=40), Females (n=98)  13-25yrs old | Hispanic (n=74),  African American (n=64) | Obesity | Pancreatic fat is elevated in Hispanic compared to African American participants.  Males had higher pancreatic fat compared to females, with pancreatic fat being correlated with VAT, hepatic fat and circulatory FFA.  No correlation between elevated pancreatic fat and β-cell function. |
| Goree et al. (2010) (7) | Female (n=154)  7-72yrs | European Americans (n=78)  African American (n=76) | ND | The African American cohort had lower basal fasting FFA conc. compared to European American cohort independent of insulin and body composition.  Fasting insulin and acute insulin response to glucose were higher in the African American cohort compared to European American cohort. |
| Burns et al. (2009) (8) | Males (n=24), Female (n=19) 13-14yrs | African American (n=22), Caucasian (n=21) | Family hx of T2D (n=26)  No family hx of T2D (n=17) | During 20% intralipid infusion, fasting insulin, C-peptide concentrations, and TAG concentrations increased comparably in both groups |
| Tushuizen et al. (2007) (9) | Males (n=36)  35-65yrs old | Caucasian | T2D (n=12)  ND (n=24) | Pancreatic and hepatic fat in T2D significantly higher compared to ND group.  Pancreatic fat correlated negatively with β-cell function but only in the ND group. |

**Ethnicity as described by authors of the study. Hx: history. ND: Non-diabetic. T2D: Type 2 diabetes.*

**References:**

1. Ladwa M, Bello O, Hakim O, Shojaee-Moradie F, Boselli ML, Charles-Edwards G, et al. Ethnic differences in beta cell function occur independently of insulin sensitivity and pancreatic fat in black and white men. BMJ Open Diabetes Res Care. 2021;9(1).
2. Tricò D, Mengozzi A, Nesti L, Hatunic M, Gabriel Sanchez R, Konrad T, et al. Circulating palmitoleic acid is an independent determinant of insulin sensitivity, beta cell function and glucose tolerance in non-diabetic individuals: a longitudinal analysis. Diabetologia. 2020;63(1): 206-218.
3. Michaliszyn SF, Bonadonna RC, Sjaarda L a, Lee S, Farchoukh L, Arslanian S a. β-Cell lipotoxicity in response to free fatty acid elevation in prepubertal youth: African American versus Caucasian contrast. Diabetes. 2013;62(8): 2917-22.
4. Tillin T, Hughes AD, Godsland IF, Whincup P, Forouhi NG, Welsh P, Sattar N, McKeigue PM, Chaturvedi N. Insulin resistance and truncal obesity as important determinants of the greater incidence of diabetes in Indian Asians and African Caribbeans compared with Europeans. Diabetes Care. 2013; 36(2): 383-393.
5. Anand SS, Tarnopolsky MA, Rashid S, Schulze KM, Desai D, Mente A, et al. Adipocyte hypertrophy, fatty liver and metabolic risk factors in south asians: The molecular study of health and risk in ethnic groups (mol-SHARE). PLoS One. 2011;6(7).
6. Lè KA, Ventura EE, Fisher JQ, Davies JN, Weigensberg MJ, Punyanitya M, et al. Ethnic differences in pancreatic fat accumulation and its relationship with other fat depots and inflammatory markers. Diabetes Care. 2011; 34(2): 485-90.
7. Goree LLT, Darnell BE, Oster RA, Brown MA, Gower BA. Associations of free fatty acids with insulin secretion and action among African-American and European- American girls and women. Obesity. 2010;18(2): 247-53.
8. Burns SF, Kelsey SF, Arslanian SA. Effects of an intravenous lipid challenge and free fatty acid elevation on in vivo insulin sensitivity in african american versus caucasian adolescents. Diabetes Care. 2009;32(2): 355-60.
9. Tushuizen ME, Bunck MC, Pouwels PJ, Bontemps S, Van Waesberghe JHT, Schindhelm RK, et al. Pancreatic fat content and β-cell function in men with and without type 2 diabetes. Diabetes Care. 2007;30(11): 2916-21.
