# Supplementary material for: Beta cell lipotoxicity in the development of type 2 diabetes: the need for species-specific understanding
Source: Front Endocrinol (Lausanne). 2023 Dec 8;14:1275835. doi: 10.3389/fendo.2023.1275835 (PMC10739424; doi:10.3389/fendo.2023.1275835)
Supplement: Supplementary file 2 [file Table_2.docx]

| **Supplementary Table 2. Changes to the transcriptome of fatty acid treated human islets.** | | | | | |
| --- | --- | --- | --- | --- | --- |
| Ref. | Treatment | Exposure  Period (hours) | No. of modified GWAS T2D candidate genes | No. of differentially expressed genes (DEG) | DEG or functional annotation for DEG |
| Hall *et al.* (2019)  (1) | 19mM glucose + 1mM C16:0 | 48 | 16 | 1,855 | **Upregulated:**  Protein export  SNARE interactions in vesicular transport |
|  |  |  |  |  | **Downregulated:**  Retinol metabolism  TGF signalling pathway  Glycolysis/  gluconeogenesis  Starch and sucrose metabolism  Fatty acid metabolism  Citrate cycle TCA cycle  Cell cycle  Inositol phosphate metabolism |
| Sargsyan *et al.* (2011) (2) | 500µM C16:0 | 0, 4, 12, 24, 168 | - | 903 | **At 4h:**  Mineral absorption |
|  |  |  |  |  | **At 12h:**  PPAR signalling pathway  Adipocytokine signalling pathway |
|  |  |  |  |  | **At 24h:**  PPAR signalling pathway  Adipocytokine signalling pathway  Glycolysis/  gluconeogenesis  Fatty acid degradation  TNF signalling pathway |
|  |  |  |  |  | **At 48h:**  Glycolysis/gluconeogenesis  Fatty acid degradation  TNF degradation  Glycerolipid metabolism  Fat digestion and absorption  Chemokine and signalling pathway  Protein digestion and absorption  Pancreatic secretion |
|  |  |  |  |  | **At 168h:**  Glycolysis/gluconeogenesis  Fatty acid degradation  Glycolipid metabolism  Fat digestion and absorption  Pancreatic secretion |
| Brun *et al.*  (2015) (3) | 400µM C16:0 | 72 | - | - | **Upregulated:** *SIRT1, MPC1, MCP2* |
|  |  |  |  |  | **Downregulated:** *MT-ATP8, GC1* |
|  | 400µM C18:1 | 72 |  |  | **Downregulated:** *MAFA, PDX-1, MT-ATP8, MT-ND5, MT-ND6, MT-C03, AGC1* |
| Cnop *et al.*  (2014) (4) | 400µM C16:0 | 48 | 11 | 1,325 | **Upregulated:**  ER stress  Cytosolic stress  Inflammation  Fatty acid metabolism  Apoptosis |
|  |  |  |  |  | **Downregulated:**  ATP production  Potassium channels  Growth/beta cell regeneration  Autophagy  Apoptosis  Transcription factors associated with beta cell function and identity (*PDX-1, MAFA, MAFB, PAX4, NEUROD1*) |
| Dayeh *et al.*  (2014) (5) | 1mM C16:0 | 48 | - | - | **Upregulated:**  *GADD45A* |
|  |  |  |  |  | **Downregulated:**  *DNMT3a, DNMT1* |
| Hall *et al.* (2014) (6) | 1mM C16:0 | 48 | 16 | 1,860 | **Upregulated:**  Aminoacyl-tRNA biosynthesis  PPAR signalling pathway  Amino sugar and nucleotide sugar metabolism |
|  |  |  |  |  | **Downregulated:**  Metabolic pathways  Glycolysis/gluconeogenesis  Arginine and proline metabolism  Fatty acid metabolism  Cell cycle  Glutathione, Butanoate, Arachidonic, pyruvate metabolism  Valine, leucine and isoleucine degradation |

*C16:0: palmitate, C18:1: oleate, ER: endoplasmic reticulum, PPAR: peroxisome proliferator activated receptor, TNF: tumor necrosis factor*

**References:**

1. Hall E, Jönsson J, Ofori JK, Volkov P, Perfilyev A, Nitert MD, et al. Glucolipotoxicity alters insulin secretion via epigenetic changes in human islets. Islets Studies. 2019; 68(10): 1965-1974.
2. Sargsyan E, Bergsten P. Lipotoxicity is glucose-dependent in INS-1E cells but not in human islets and MIN6 cells. Lipids Health Dis. 2011;10.
3. Brun T, Li N, Jourdain AA, Gaudet P, Duhamel D, Meyer J, et al. Diabetogenic milieus induce specific changes in mitochondrial transcriptome and differentiation of human pancreatic islets. Hum Mol Genet. 2015;24(18): 5270-84.
4. Cnop M, Abdulkarim B, Bottu G, Cunha DA, Igoillo-Esteve M, Masini M, et al. RNA sequencing identifies dysregulation of the human pancreatic islet transcriptome by the saturated fatty acid palmitate. Diabetes. 2014;63(6): 1978-93.
5. Dayeh T, Volkov P, Salö S, Hall E, Nilsson E, Olsson AH, et al. Genome-Wide DNA Methylation Analysis of Human Pancreatic Islets from Type 2 Diabetic and Non- Diabetic Donors Identifies Candidate Genes That Influence Insulin Secretion. PLoS Genet. 2014;10(3).
6. Hall E, Volkov P, Dayeh T, Bacos K, Rönn T, Nitert MD, et al. Effects of palmitate on genome-wide mRNA expression and DNA methylation patterns in human pancreatic islets. BMC Med. 2014;12(1).
